# Validation study of the Japanese version of the faecal incontinence quality of life scale

**DOI:** 10.1111/j.1463-1318.2011.02558.x

**Published:** 2011-02-02

**Authors:** H Ogata, T Mimura, K Hanazaki

**Affiliations:** * Department of Surgery, Kochi Medical School, Kochi UniversityKochi, Japan; † Pelvic Floor Center, Kochi Medical School, Kochi UniversityKochi, Japan

**Keywords:** Faecal incontinence, quality of life, faecal incontinence quality of life, scale, validity, reliability

## Abstract

**Aim:**

The aim of the present study was to conduct a psychometric validation of the Japanese version of the FIQL (JFIQL).

**Method:**

A retrospective analysis of data from the JFIQL was conducted. Wexner scores and Faecal Incontinence Severity Index (FISI) scores were collected prospectively in patients with faecal incontinence who visited our centre between 2008 and 2009. For convergent validity, the JFIQL scores were compared with stages on the Wexner scale for lifestyle alteration. To evaluate reliability, Cronbach's alpha was calculated for internal consistency, whereas a test–retest study was performed to evaluate reproducibility. In assessing responsiveness, JFIQL scores before and after treatments were compared in patients whose FISI scores decreased by ≥ 50%.

**Results:**

Convergent validity and internal consistency were determined in 70 patients (49 women; median age 68.5 years). The JFIQL scores were significantly associated with lifestyle alteration stages on the Wexner scale, demonstrating convergent validity in all four domains and the generic score. Cronbach's alpha was > 0.7 for generic scores and all domains except Embarrassment. The intraclass correlations for the 27 patients available for the test–retest study were > 0.7 for generic scores and all domains except Embarrassment. The median JFIQL score improved significantly after treatment in the 23 patients whose FISI scores decreased ≥ 50%, indicating good responsiveness in all four domains and the generic score.

**Conclusion:**

The JFIQL has been validated and is now ready for use in evaluating the symptom-specific quality of life in Japanese patients with faecal incontinence.

**What is new in this paper:**

This is the first study validating the Japanese version of the FIQL (JFIQL). The JFIQL was validated not only for convergent validity and reliability, but also for responsiveness, which has never been addressed before. We also validated a generic JFIQL score in addition to scores for four specific domains.

## Introduction

Faecal incontinence impairs quality of life (QOL) [1], causing embarrassment and psychological distress, as well as limiting daily activities. In order to choose an optimal therapy and evaluate the efficacy of treatment, individual symptoms and the QOL must be assessed as accurately and objectively as possible.

In our institution, the Cleveland Clinic Florida Faecal Incontinence score, the so-called Wexner score [2], the Faecal Incontinence Severity Index (FISI) [3] and the Faecal Incontinence Quality of Life Scale (FIQL) [4] are used to evaluate symptom severity and QOL in patients with faecal incontinence.

In 2000, Rockwood *et al.* [4] published the FIQL, which was specifically designed to evaluate the QOL of patients with faecal incontinence and was validated in English. Since then, the FIQL has been translated and validated in several languages, including French [5], Portuguese [6], Italian [7], Spanish [8] and Turkish [9]. The aim of the present study was to develop a Japanese version of the FIQL (JFIQL) and to assess its psychometric properties in Japanese patients with faecal incontinence.

## Method

### Patients

Data for the JFIQL were collected prospectively after the questionnaire had been self-administered by consecutive patients presenting to the Pelvic Floor Center, Kochi Medical School Hospital, with a chief complaint of faecal incontinence between September 2008 and August 2009. Patients’ symptoms were also evaluated with a structured questionnaire that yielded the Wexner score and FISI. Patients were evaluated using the JFIQL, Wexner and FISI on another two occasions: one when an anorectal physiology examination was performed and another after some patients had received treatment for their faecal incontinence.

### JFIQL

The FIQL comprises 29 questions in four domains, namely Lifestyle (10 items), Coping/Behaviour (nine items), Depression/Self-perception (seven items) and Embarrassment (three items). Each domain of the JFIQL was scored according to the original publication [4]. In the present study, the equation used to calculate the score for the Coping/Behaviour domain was corrected, adopting Q3-c instead of Q3-d, which seems to have been a typographical error in the original paper [10]. A generic score, which was not used in the original English version, was calculated as an average of all 29 items. This generic score was used as an index of the general faecal incontinence-specific QOL.

The English FIQL was translated into Japanese by one of the authors (TM), who is fluent in both Japanese and English and has considerable expertise in the area of functional bowel disorders [11]. Some modifications were made to adapt the English version to Japanese culture and linguistics. First, the term ‘to church’ in Q2-d was replaced by ‘shopping’ because going to church is not a customary practice in Japan. Second, the answers in section Q3 were changed from referring to ‘degree’ to ‘frequency’, which is more natural for Japanese people. This modification also appears in the Spanish version of the FIQL [8]. The JFIQL is provided in the Appendix S1.

### Validation methods

The psychometric properties of the JFIQL were determined in terms of convergent validity, reliability and responsiveness, as described below. Analysis was conducted for the four domains and for the generic score.

### Validity

To test the convergent validity of the JFIQL, JFIQL scores were compared with the QOL component of the Wexner scale that related to lifestyle alterations. Both scores were determined from data collected at the patient's first visit. Lifestyle alterations on the Wexner scale are classified into five stages depending on the frequency of lifestyle changes due to faecal incontinence [2].

Mean JFIQL scores for each of the Wexner lifestyle alteration stages were calculated and compared using one-way ANOVA to identify any trends among the five stages. A positive association between JFIQL scores and Wexner lifestyle alterations can be taken as evidence of convergent validity.

### Reliability

Internal consistency and reproducibility were investigated to evaluate the reliability of the instrument. Internal consistency examines the complementary nature of items by searching for contradictions and measurement errors. To evaluate internal consistency, Cronbach's alpha was calculated for the generic score and all four domains. A high positive value for Cronbach's alpha (i.e. ≥ 0.70) suggests that the JFIQL measures consistently.

To evaluate reproducibility, a test–retest study was performed by comparing JFIQL scores obtained at the time of the patient's first visit with those obtained at the second visit, when anorectal physiology examinations were undertaken without any interventions applied between the two visits. Comparisons were made using intraclass correlation analysis and a high positive correlation coefficient (i.e. ≥ 0.70) can be taken as evidence of reproducibility.

### Responsiveness

To assess the sensitivity of the JFIQL in detecting changes in QOL after some treatment, its responsiveness was evaluated. For this purpose, JFIQL scores obtained at the time of the patient's first visit were compared with those obtained in patients whose symptoms of faecal incontinence improved significantly after some treatment. Significant symptomatic improvement was defined as a reduction in the FISI of ≥ 50%.

### Statistical analysis

Data were regarded as parametric and are expressed as the mean ± SD. Statistical analyses were performed using PASW Statistics version 18 (SPSS Japan Inc., Tokyo, Japan). One-way ANOVA, Cronbach's alpha, intraclass correlations, and paired *t*-tests were conducted as appropriate. *P* < 0.05 was considered significant.

### Ethics

Because this questionnaire survey was conducted as a part of our clinical practice and was needed to provide the best possible care for the patients, our Institutional Research Board did not require us to obtain ethical approval for the study. However, written informed consent was obtained from all patients who participated in the study at the time of their initial visit so that their clinical data could be used for any clinical study so long as their privacy was not jeopardized.

## Results

**Figure 1:**
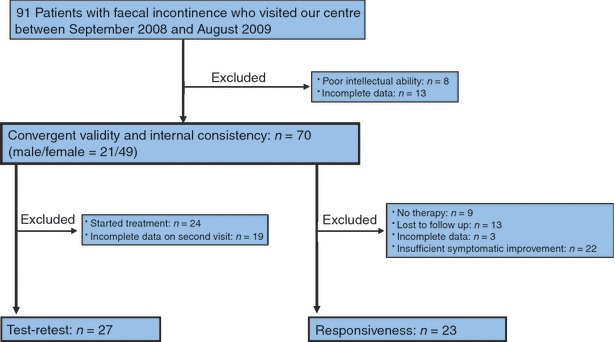
Flow diagram of patients included in the study and those excluded from analysis for various reasons.

During the study period, 91 patients presented at our centre with the chief complaint of faecal incontinence. Twenty-one were excluded owing to lack of data. (Fig. 1). The characteristics of these patients are given in Table 1.

**Table 1:** Characteristics of the 70 patients with faecal incontinence

Age (years)	68.5 ± 18.9
Gender, M/F, *n*	21/49
Duration of FI (months)	57.1 ± 99.6
FISI	17.0 ± 1.3
Wexner	8.8 ± 0.6
Generic score of the JFIQL	3.0 ± 0.1

Unless indicated otherwise, data show the mean ± SD.

FI, faecal incontinence; FISI, Faecal Incontinence Severity Index; Wexner, Cleveland Clinic Florida Faecal Incontinence score; JFIQL, Japanese version of Faecal Incontinence Quality of Life Scale.

### Convergent validity

Analysis using one-way ANOVA indicated a significant association between lifestyle changes due to faecal incontinence for the Wexner score and the generic JFIQL score and the scores in all four domains. Specifically, the more frequent the lifestyle alterations using the Wexner score, the lower the JFIQL scores for each of the four domains and the generic score. The *P* value was < 0.001 for scores in Lifestyle, Coping/Behaviour and Embarrassment domains, as well as for the generic score, whereas it was 0.04 for the score in the Depression/Self-perception domain.

### Reliability

The internal consistency of the JFIQL in the 70 patients, evaluated using Cronbach's alpha, was found to be 0.92 for Lifestyle, 0.87 for Coping/Behaviour, 0.83 for Depression/Self-perception, 0.68 for Embarrassment, and 0.95 for the generic score. These results indicate that the JFIQL measures consistently for the generic score and across all domains except Embarrassment.

In the test–retest study, data were available for 27 of 70 patients (39%) at the second visit (Fig. 1). The mean interval between the first and second visits was 29 ± 14 days. Intraclass correlation coefficients for the 27 patients are given in Table 2. There was a good correlation between JFIQL scores obtained on the first and second visits for the generic score and across all domains except Embarrassment.

**Table 2:** Reproducibility (test–retest study)

Domains (no. items)	Score at first visit	Score at second visit	Intraclass correlation coefficient
Lifestyle (10)	3.00 ± 0.84	3.02 ± 0.82	0.76
Coping/Behaviour (9)	2.45 ± 0.77	2.68 ± 0.75	0.74
Depression/Self-perception (7)	2.90 ± 0.70	3.15 ± 0.75	0.72
Embarrassment (3)	2.37 ± 0.71	2.67 ± 0.66	0.59
Generic score (29)	2.74 ± 0.63	2.97 ± 0.64	0.77

Data are the mean ± SD.

### Responsiveness

Of the 70 patients in the study, 23 achieved significant symptomatic improvement and served as the subjects for the responsiveness study (Fig. 1). In these 23 patients, the mean generic JFIQL score improved significantly after treatment from 2.55 to 3.41 (Table 3). Similar significant improvements were observed in JFIQL scores across all four domains.

**Table 3:** Responsiveness

	Score before treatment	Score after treatment	*P* value (paired *t*-test)
Lifestyle	3.00 ± 0.98	3.83 ± 0.78	< 0.001
Coping/Behaviour	2.22 ± 0.85	3.22 ± 0.79	< 0.001
Depression/Self-perception	2.93 ± 0.79	3.66 ± 0.65	< 0.001
Embarrassment	2.22 ± 1.03	3.33 ± 0.55	0.006
Generic score	2.55 ± 0.83	3.41 ± 0.67	< 0.001

Data are the mean ± SD.

## Discussion

The present study provides sufficient evidence supporting the reliability and validity of the JFIQL, not only in convergent validity and reliability, but also in responsiveness, which has not been addressed by other validation studies of the FIQL. The present study also validated the generic JFIQL score in addition to scores for each of the four domains.

We sought permission from Dr Rockwood, the first author of the original FIQL, to conduct a validation study of the JFIQL, but he stated that permission was not required because the FIQL ‘is in the public domain and freely available to anyone and everyone for whatever use they choose’.

Regarding convergent validity, there is no gold standard faecal incontinence-specific QOL questionnaire that can be compared with the FIQL. Some papers [6,8,9], including the original FIQL study [4], have compared the FIQL with the SF-36 [12] and reported a significant correlation between the two. However, the correlation coefficients reported were rather low, ranging between 0.28 and 0.65, indicating that, in fact, there was not a good correlation between the FIQL and SF-36. This is quite natural because the SF-36 is not a symptom-specific questionnaire for faecal incontinence.

In the present study, lifestyle alterations on the Wexner scale were used as a comparison to determine the convergent validity of the JFIQL because the lifestyle alterations on the Wexner scale are an indicator of changes in QOL specific to faecal incontinence. Although only one item on the Wexner scale was used, there was a significant association between lifestyle alterations on the Wexner scale and JFIQL scores, confirming the convergent validity of the JFIQL for faecal incontinence.

A strong internal consistency was demonstrated for the generic score and across all domains except Embarrassment. Exceptions for the Embarrassment domain have also been reported for versions of the FIQL in other languages [5–9], with the discussion centring on the fact that the Embarrassment domain contains only three items, a much smaller number than in the other three domains. However, the real reason for the exceptions noted for Embarrassment may be that item Q2-l is not a suitable question for inclusion in this domain. Item Q2-l asks about symptoms only, whereas the other two items in this domain specifically ask about feeling embarrassed. In order to confirm our reasoning, we performed ‘If-item-deleted’ analysis. The results of this analysis indicated that Q2-l was not consistent with Q3-a and Q3-e for Embarrassment; specifically, Cronbach's alpha without Q2-l was 0.73, much higher than the values of 0.57 and 0.41 obtained if Q3-a and Q3-e were omitted, respectively.

Strong reproducibility was demonstrated for the generic score and all four domains except Embarrassment despite the relatively long interval of 29 ± 14 days between the first and second visits in the present study. Although this long interval is due, in part, to the retrospective nature of our study, it may more likely reflect the actual situation of clinical practice than a shorter interval of 7–10 days, which has been used in prospective studies of the instrument in other languages (Table 4) [5,6,8,9]. The short interval could overestimate the test–retest reliability because, at the time of the second test, patients may be able to recall what they had answered in the first test.

**Table 4:** Comparison of published data with regard to intraclass correlation coefficients

	Rullier *et al.*[8] (French)	Minguez *et al.*[11] (Spanish)	Dedeli *et al.*[12] (Turkish)	Present study (Japanese)
Lifestyle	0.93	0.92	0.94	0.76
Coping/Behaviour	0.86	0.90	0.90	0.74
Depression/Self-perception	0.87	0.85	0.88	0.72
Embarrassment	0.80	0.74	0.76	0.59
Generic score	–	–	0.97	0.77
Test–retest interval (days)	7	7–10	7–10	29 ± 14
Patients in the study, *n*	100	111	50	27

The relatively low intraclass correlation coefficient for the Embarrassment domain obtained in the present study has also been reported by studies of the instrument in other languages (Table 4) [5,8,9]. As discussed above for internal consistency, this may be due to the small number of items in the Embarrassment domain. However, another possibility is that any feelings of embarrassment felt by patients were reduced after the first visit, in which patients were able to discuss their perceived shameful symptoms of faecal incontinence with their doctor for the first time. This discussion may have resulted in an unintentional reduction in their feelings of embarrassment at the time of the second visit.

Good responsiveness was confirmed in the present study, with JFIQL scores having increased significantly in accordance with marked symptomatic improvements. Although Kwon *et al.* [13] emphasized the importance of responsiveness in a QOL questionnaire, this aspect has not been analysed by the studies performed in other languages [5–9], probably because the duration of those studies was not long enough to include patient treatment.

There are two major limitations of the present study. First, this is a retrospective study, which resulted in many dropouts because of incomplete data and/or the initiation of treatment prior to the retest. Second, a formal linguistic validation was not performed using a translation–back translation method or a linguistic consensus board.

The FIQL is the best symptom-specific questionnaire available at present and deserves to be called the gold standard for the evaluation of the QOL of patients with faecal incontinence because it has been validated in several languages and is used more and more frequently in many high-quality studies. The utilization of the FIQL makes it easy to compare international studies on faecal incontinence and enables us to conduct international multicentre studies in several languages. We hope that the FIQL will be translated and validated in more languages.

## Supporting Information

**Appendix S1.** The Japanese version of the Faecal Incontinence Quality of Life Scale (JFIQL).
