# Supplementary material for: Validation study of the Japanese version of the faecal incontinence quality of life scale
Source: Colorectal Dis. 2011 Feb 2;14(2):194–9. doi: 10.1111/j.1463-1318.2011.02558.x (PMC3440588; doi:10.1111/j.1463-1318.2011.02558.x)
Supplement: Supplementary file 1 [file codi0014-0194-SD1.doc]

**Appendix (Online-Only)**

**日本語版Fecal Incontinence Quality of Life Scale （JFIQL）**

**Q1: 全体的に考えて、あなたの健康状態はいかがですか。**

　　　1．素晴らしい

2．とても良い

3．良い

4．あまり良くない

5．悪い

**Q2: 以下の各項目について、便失禁のために当てはまる頻度を１～４から選んでください。**

**その項目が自分に無関係な質問であったり、たとえ関係してもそれが便失禁とは無関係の場合は**

**「該当せず」にマルをしてください。**

| Q2. 便失禁のために | **ほぼ常に** | **時々** | **まれに** | **全くない** | **該当せず** |
| --- | --- | --- | --- | --- | --- |
| a.外出したくない | 1 | 2 | 3 | 4 | □ |
| b.友達の家に行くのを避ける | 1 | 2 | 3 | 4 | □ |
| c.外泊を避ける | 1 | 2 | 3 | 4 | □ |
| d.映画や買い物に出かけるのが難しい | 1 | 2 | 3 | 4 | □ |
| e.外出前の食事の量を減らす | 1 | 2 | 3 | 4 | □ |
| f.外出中はなるべくトイレの近くに居る | 1 | 2 | 3 | 4 | □ |
| g.排便のタイミングに合わせて日頃の計画を立てる | 1 | 2 | 3 | 4 | □ |
| h.旅行を避ける | 1 | 2 | 3 | 4 | □ |
| i.トイレに間に合わないのが心配 | 1 | 2 | 3 | 4 | □ |
| j.排便に関してコントロールできない気がする | 1 | 2 | 3 | 4 | □ |
| k.排便の時にトイレまで間に合わない | 1 | 2 | 3 | 4 | □ |
| l.知らないうちに便が漏れている | 1 | 2 | 3 | 4 | □ |
| m.トイレのそばに居ることによって便失禁を防ごうとしている | 1 | 2 | 3 | 4 | □ |

**Q3: 以下の各項目について、便失禁のために当てはまる頻度を１～４から選んでください。**

**その項目が自分に無関係な質問であったり、たとえ関係してもそれが便失禁とは無関係の場合は**

**「該当せず」にマルをしてください。**

| Q3. 便失禁のために | **ほぼ常に** | **時々** | **まれに** | **全くない** | **該当せず** |
| --- | --- | --- | --- | --- | --- |
| a.恥ずかしいと感じる | 1 | 2 | 3 | 4 | □ |
| b.やりたいと思った事ができない | 1 | 2 | 3 | 4 | □ |
| c.便を漏らすのではないかと心配だ | 1 | 2 | 3 | 4 | □ |
| d.気分が落ち込む | 1 | 2 | 3 | 4 | □ |
| e.他人が、私がウンコ臭いと思うのではないかと心配だ | 1 | 2 | 3 | 4 | □ |
| f.自分が健康ではないと感じる | 1 | 2 | 3 | 4 | □ |
| g.人生が楽しくない | 1 | 2 | 3 | 4 | □ |
| h.自分がしたいと思う回数のセックスができない | 1 | 2 | 3 | 4 | □ |
| i.自分が他人と違う気がする | 1 | 2 | 3 | 4 | □ |
| j.便を漏らすのではないかという不安が常に頭の中にある | 1 | 2 | 3 | 4 | □ |
| k.セックスするのが不安だ | 1 | 2 | 3 | 4 | □ |
| l.飛行機や電車で旅行する事を避ける | 1 | 2 | 3 | 4 | □ |
| m.外食を避ける | 1 | 2 | 3 | 4 | □ |
| n.知らないところへ初めて行ったら、必ずトイレの場所を  確認する | 1 | 2 | 3 | 4 | □ |

**Q4. 過去1ヶ月の間、何もやる気が起きないくらいに、悲しかったり、がっかりしたり、**

**たくさんの問題を抱えましたか。**

1. 全くその通り（全ての事をあきらめる程ひどかった）

2. 非常にその通り

　　　3. とてもその通り

4. ある程度はその通り（気になる程度であった）

5. 少しだけその通り

6. 全くそんなことはなかった（とても快適だった）

各群および総合評価の点数算出方法

| Q１は５段階で、Q２と３は４段階で、Q４は6段階で評価する。  Q２、３、４は、点数が低いほどQOLが低いことを意味するが、Q１のみは点数が低いほどQOLが高いことを意味するため、Q１のみ集計時に点数を逆転する必要がある。  各群のスコアは平均値で示す。すなわち、各群の項目の全ての点数を合計した後、その群の項目数で割る。  「該当せず」は欠損データとして扱い、平均値の算出対象から除外する。  １群．生活スタイル（Lifestyle）, 10項目: Q2a, Q2b, Q2c, Q2d, Q2e, Q2g, Q2h, Q3b, Q3l, Q3m  ２群．対処/日常行動（Coping/Behavior）,9項目: Q2f, Q2i, Q2j, Q2k, Q2m, Q3c, Q3h, Q3j, Q3n  ３群．憂鬱感/自己認識（Depression/Self Perception）, 7項目: Q1, Q3d, Q3f, Q3g, Q3i, Q3k, Q4  ４群．羞恥心（Embarrassment）, 3項目: Q2l, Q3a, Q3e  総合評価（Generic score）, 全29項目 |
| --- |

**Back translated English Version of the JFIQL**

**Q1: In general, would you say your health is:**

1 □ Excellent

2 □ Very Good

3 □ Good

4 □ Fair

5 □ Poor

**Q2: For each of the items, please indicate how much of the time the issue is a concern for you**

**due to accidental bowel leakage. (If it is concern for you for reasons other than**

**accidental bowel leakage then check the box under Not Apply, (N/A).)**

| Q2. Due to accidental bowel leakage: | Most of  the Time | Some of  the Time | A Little of  the Time | None of  the Time | N/A |
| --- | --- | --- | --- | --- | --- |
| a. I am afraid to go out | 1 | 2 | 3 | 4 | □ |
| b. I avoid visiting friends | 1 | 2 | 3 | 4 | □ |
| c. I avoid staying overnight away from home | 1 | 2 | 3 | 4 | □ |
| d. It is difficult for me to get out and do things  like going to a movie or shopping | 1 | 2 | 3 | 4 | □ |
| e. I cut down on how much I eat before I go out | 1 | 2 | 3 | 4 | □ |
| f. Whenever I am away from home, I try to stay  near a restroom as much as possible | 1 | 2 | 3 | 4 | □ |
| g. It is important to plan my schedule (daily  activities) around my bowel pattern | 1 | 2 | 3 | 4 | □ |
| h. I avoid traveling | 1 | 2 | 3 | 4 | □ |
| i. I worry about not being able to get to the  toilet in time | 1 | 2 | 3 | 4 | □ |
| j. I feel I have no control over my bowels | 1 | 2 | 3 | 4 | □ |
| k. I can't hold my bowel movement long  enough to get to the bathroom | 1 | 2 | 3 | 4 | □ |
| l. I leak stool without even knowing it | 1 | 2 | 3 | 4 | □ |
| m. I try to prevent bowel accidents by staying  very near a bathroom | 1 | 2 | 3 | 4 | □ |

**Q3: Due to accidental bowel leakage, indicate how much of the time the issue is a concern for you**

**for each of the following items. (If it is a concern for you for reasons other than accidental bowel**

**leakage then check the box under Not Apply, (N/A).)**

| Q3. Due to accidental bowel leakage: | Most of  the Time | Some of  the Time | A Little of  the Time | None of  the Time | N/A |
| --- | --- | --- | --- | --- | --- |
| a. I feel ashamed | 1 | 2 | 3 | 4 | □ |
| b. I can not do many of things I want to do | 1 | 2 | 3 | 4 | □ |
| c. I worry about bowel accidents | 1 | 2 | 3 | 4 | □ |
| d. I feel depressed | 1 | 2 | 3 | 4 | □ |
| e. I worry about others smelling stool on me | 1 | 2 | 3 | 4 | □ |
| f. I feel like I am not a healthy person | 1 | 2 | 3 | 4 | □ |
| g. I enjoy life less | 1 | 2 | 3 | 4 | □ |
| h. I have sex less often than I would like to | 1 | 2 | 3 | 4 | □ |
| i. I feel different from other people | 1 | 2 | 3 | 4 | □ |
| j. The possibility of bowel accidents is  always on my mind | 1 | 2 | 3 | 4 | □ |
| k. I am afraid to have sex | 1 | 2 | 3 | 4 | □ |
| l. I avoid traveling by plane or train | 1 | 2 | 3 | 4 | □ |
| m. I avoid going out to eat | 1 | 2 | 3 | 4 | □ |
| n. Whenever I go someplace new, I  specifically locate where the bathrooms are | 1 | 2 | 3 | 4 | □ |

**Q4. During the past month, have you felt so sad, discouraged, hopeless, or had so many**

**problems that you wondered if anything was worthwhile?**

1 □ Extremely So – To the point that I have just about given up

2 □ Very Much So

3 □ Quite a Bit

4 □ Some – Enough to bother me

5 □ A Little Bit

6 □ Not At All – Very comfortable

Scale Scoring

| Scales range from 1 to 5 in Q1, from 1 to 4 in Q2 & Q3, and from 1 to 6 in Q4, with a 1 indicating a lower functional status of quality of life except Q1. Therefore, Q 1 is to be reverse coded.  Domain scores are the average (mean) response to all items in the domain (e.g., add the responses to all questions in a domain together and then divide by the number of items in the domain. Not Apply is coded as a missing value in the analysis for all questions.)  Domain 1. Lifestyle, ten items: Q2a, Q2b, Q2c, Q2d, Q2e, Q2g, Q2h, Q3b, Q3l, Q3m  Domain 2. Coping/Behavior, nine items: Q2f, Q2i, Q2j, Q2k, Q2m, Q3c, Q3h, Q3j, Q3n  Domain 3. Depression/Self Perception, seven items: Q1, Q3d, Q3f, Q3g, Q3i, Q3k, Q4  Domain 4. Embarrassment, three items: Q2l, Q3a, Q3e  Generic score, all the twenty-nine items |
| --- |
